# Enhanced performance of non-thermal plasma coupled with TiO_2_/GAC for decomposition of chlorinated organic compounds: influence of a hydrogen-rich substance

**DOI:** 10.1186/s40201-014-0119-1

**Published:** 2014-09-16

**Authors:** Kamaleddin Abedi, Farshid Ghorbani-Shahna, Babak Jaleh, Abdolrahman Bahrami, Rasoul Yarahmadi

**Affiliations:** Department of occupational health engineering, Research center for health services, Hamedan University of Medical Sciences, Hamedan, Iran; Department of physics, Bu-Ali Sina University, Hamedan, Iran; Department of occupational health, Occupational health research center, School of public health, Iran University of Medical Sciences, Tehran, Iran

**Keywords:** CVOCs removal, NTP-catalysis, TiO_2_, Granular activated carbon, Hydrogen-rich source

## Abstract

**Background:**

No study was found in the literature on the combination of TiO_2_/GAC catalyst and non-thermal plasma for chlorinated volatile organic compounds abatement in air. This paper presents this hybrid process for the decomposition of chloroform (as a target compound) using a multi-pin to plate discharge reactor. The experiments were performed using a high frequency pulsed transformer as the power supply system to examine the effect of SIE, frequency, as well as initial concentration on the chloroform removal efficiency (RE). Toluene was added as a hydrogen-rich source to shift the reactions into the formation of environmentally desirable products.

**Results:**

RE of around 60% was observed with the NTP-alone process at the highest possible SIE (3000 J L^−1^), while it rocketed up to 100% (total oxidation) in the presence of TiO_2_/GAC at SIE of 1000 J L^−1^. About 100% O_3_ destruction over TiO_2_/GAC and both adsorption and catalytic activities of GAC may be considered as the reasons for better performance of the hybrid process. Toluene feeding diminished the chlorinated by-products such as Cl_2_ and TCE significantly. The selectivity towards CO_2_ was noticed to enhance noticeably, when both catalyst and toluene were introduced, regardless of the input concentration.

**Conclusions:**

Our findings suggest that the hybrid of NTP with TiO_2_/GAC will highly be effective in the abatement of chloroform, and the addition of toluene will successfully decline harmful chlorinated by-products.

## Background

Most of the hazardous air pollutants listed by environmental protection agencies are volatile organic compounds (VOCs) particularly chlorinated hydrocarbons [1-3]. Prolonged exposure to chlorinated VOCs (CVOCs) and their emission into atmosphere has raised considerable public concern due to the fact that most of these pollutants can lead to harmful effects on human and some of them are known to be carcinogenic [4-6]. Moreover, most of these compounds persist a long time in the environment [7]. However, they are still widely used as solvents, refrigerants, etc. in industries and workplaces. Therefore, the removal of these toxic substances is an essential issue in environmental emissions, which requires efficient and low cost technologies. Due to the high energy consumption, conventional techniques are almost ineffective particularly when the pollutant concentrations are less than 100 ppm [8]. Among the new techniques, non-thermal plasma (NTP) seems to be a very attractive and promising method, and several researchers have investigated the destruction of CVOCs using NTP, indicating that it can highly be effective in the decomposition of these compounds [9-13]. However, wasting energy as heat is still a fundamental issue in all kind of NTP reactors [14]. Previous studies have demonstrated that using a pulsed power supply is highly beneficial for both enhancing removal and energy efficiency as compared to DC- or AC-energized systems [14-18]. Furthermore, due to some limitations with the NTP-alone technology, special attention has recently been given to a hybrid use of pulsed NTP and heterogeneous catalysis, and previous works have shown that combining a metal oxide catalyst with NTP can be a preferred option [19-21]. But, because of low ability of metal oxide catalysts to adsorb organic compounds, owing to their polar structure, coating them on an adsorbent seems to enhance their efficiency resulting from the higher surface area and thereby higher residence time, yielding more oxidation and reduction rates [22]. Granular activated carbon (GAC) appears to be an extremely good adsorbent for the most of organic compounds.

In the present study, chloroform was chosen as the target CVOC which is widely used in industries, and despite its significant harmful effects, decomposition of this compound has rarely been reported in the scientific literature by plasma [11,23,24] and catalytic plasma [25]. In order to enhance catalytic reactions, GAC was employed in this work as the substrate for TiO_2_ catalyst to improve the degradation efficiency of chlorinated compounds. According to previous studies, the production of undesirable by-products such as Cl_2_ and COCl_2_ during the conversion of CVOCs can be inevitable in particular when their molecular structure contain more chlorine than hydrogen atoms [26]. Introducing a hydrogen-rich molecule such as toluene in the gas stream seems to lead the reactions to the formation of desirable chlorine containing products which are water-soluble and can be conveniently removed using a wet scrubber. Toluene was therefore fed along with chloroform to the gas stream in this study to promote the selectivity towards favorable by-products. Hence, the aim of this study was to investigate the effect of SIE, catalyst incorporation, as well as toluene addition on the chloroform removal efficiency, with special focusing on diminishing harmful by-products.

## Methods

### Reagents

Chloroform, toluene, 2-propanol, and acetyl-acetone were purchased from Merck (Germany). They were all in analytical grade with purity higher than 99.9%, 99.9%, 99.8%, and 99%, respectively. Tetra-n-butyl titanate with purity higher than 98% was supplied from Fluka (Switzerland). Granular activated carbon (with a mean diameter of 1.5-2 mm) with highest purity available (extra pure) was also obtained from Merck (Germany).

### Catalyst preparation and characterization

The catalyst was prepared by a dip-coating process using sol–gel technique. The detailed procedure has been discussed in Ref. [27]: 0.34 ml of tetra-n-butyl titanate was dissolved in a 6.1068 ml of 2-propanol and stirred for 1 h at room temperature, followed by the addition of acetyl-aceton (0.102 ml) and stirred again for 2.5 h at room temperature. By falling 2 or 3 droplets of HNO_3_ in 1.8 ml of H_2_O, an acidic solution with pH = 2 was resulted, which added completely to the former sol. The obtained mixture was then heated on a hot plate under reflux being placed in a water bath and stirred for 8 h at 75°C, resulting in a yellow transparent gel. Strict control of the reaction temperature is needed at this stage to avoid instant gelation. 0.125 grams of granular activated carbon was added in the resulting mixture (3 grams of GAC per 200 ml of sol [28]) and then subjected to vibrate in an ultrasonic bath for 0.5 h. The samples were then filtered, dried at room temperature, and calcined for 4 h at 300°C. Finally, the TiO_2_/GAC was obtained.

### Experimental system

**Figure 1:**
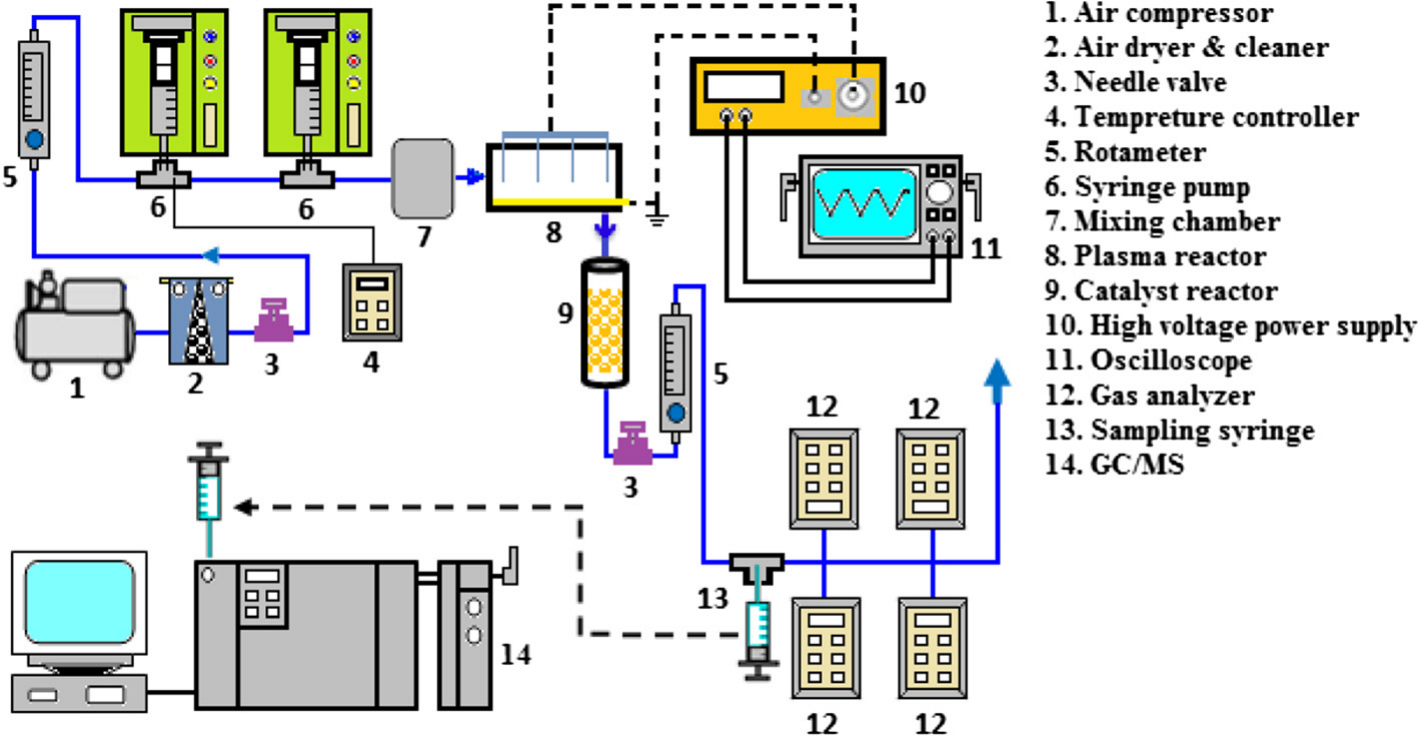
**Schematic diagram of experimental setup.**

The schematic diagram of the experimental set-up is shown in Figure 1. The experimental set-up consisted of an air supply system, a flow control and measuring unit, an injection unit, a corona discharge reactor, a catalyst reactor, an electric power supply, and some gas monitoring apparatus as shown in Figure 1. Normal air was supplied by using an air compressor with two pressure regulators. The air then passed through a mixed ceramic-activated carbon filter to be cleaned for water vapor and organic air pollutants and delivered into a mixing chamber, from which it could reach the plasma reactor. The total volumetric flow rate of gas stream was controlled to be in a range of 0.2-1 L min^−1^. The corresponding residence time was then varied from 1 to 6 s. The gas flow rate was kept at a desirable value utilizing two control valves. All the experiments were carried out at atmospheric pressure and normal temperature. Two motor driving syringe pumps, JMS SP-510 (Japan), were used to inject the liquid substances (chloroform, and toluene) into the air stream at the liquid injection port, where the temperature was kept at 40°C to facilitate the vaporization of the volatile components. The injection rate of the liquid was calculated so that the initial concentration of the contaminants in the air stream was in a desirable range (100–700 ppm). After passing through the mixing chamber, polluted gas was fed into the plasma reactor. A high voltage pulse generator (HVPL-30 OCS Haiden laboratory inc.) with peak-to-peak voltage pulses in the range of 0-30 kV and a repetition frequency of 1-20 kHz was employed to generate NTP. A flow meter was connected before and after the plasma reactor to determine the influent and effluent stream. Finally, the gas stream leaving the reactor passed through a small home-made chamber, making it possible to monitor some inorganic by-products such as CO_x_ and NO_x_ directly. The experimental set-up was carefully inspected to be completely gastight.

### Reactors configurations and electrical measurements

**Figure 2:**
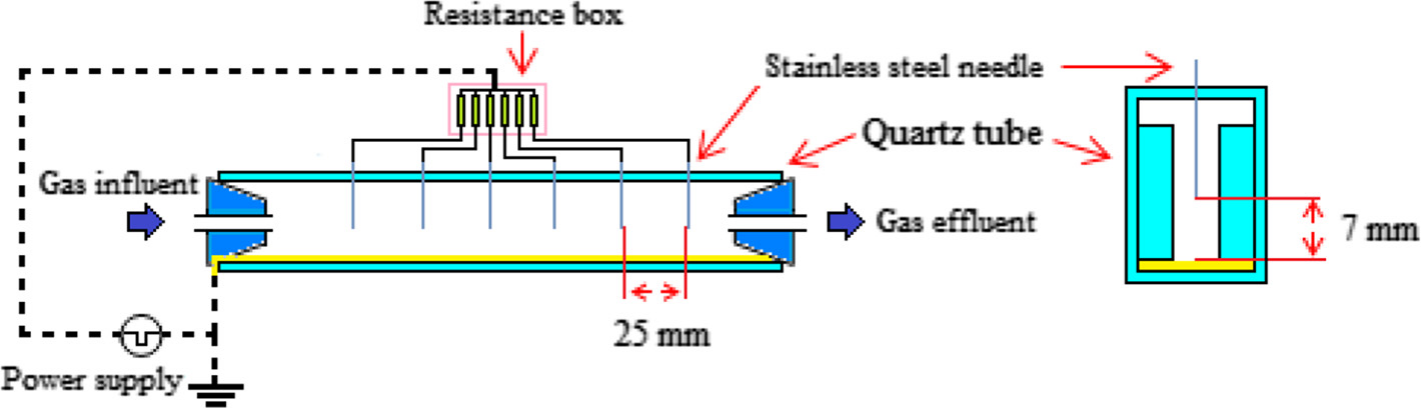
**Schematic diagram of NTP reactor.**

A rectangular corona discharge reactor was constructed of quartz with 20 × 15 × 200 mm dimensions as shown in Figure 2. An aluminum foil (200 mm length) was used as the inner electrode being placed along the floor of the reactor acting as the ground electrode. Furthermore, 6 thin stainless steel needles (each with an outer diameter of 1 mm, and a total length of 50 mm) were situated above the reactor perpendicularly against the aluminum plate acting as the discharge electrode. Then the discharge gap between ground and discharge electrodes was set to be 7 mm. The current was divided equally between electrodes using a resistance box. Small pieces of quartz were pasted inside the reactor near pin electrodes creating small tunnels along the rectangular tube so that the air stream was subjected to flow through these tunnels. This made the NTP more effective since the pollutant molecules were forced to pass as close as possible to energetic electrons. The discharge volume then became smaller and was estimated to be around 20 ml. A cylindrical glass tube, also made up of quartz, with an inner diameter of 15 mm and 100 mm length was used as catalyst reactor. About 3 grams of catalyst was placed inside the tube each time between 2 stainless steel screens. Small amounts of glass wool were also positioned in both ends of the tube to prevent particles escaping through the screens.

**Figure 3:**
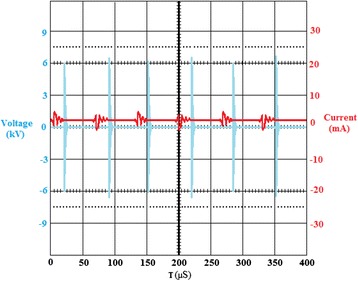
**Typical voltage and current waveforms at applied voltage of 8 kV.**

High frequency and high voltage (1–20 kHz, 8–22 kV) was applied between the electrodes to generate non-thermal plasma. Both the applied voltage and frequency were adjustable so that the plasma conditions could be optimized. A positive and negative pulsed voltage with a rising time of 200 ns and a duration time of 2 μs was applied to charge the discharge electrode. Typical voltage and current waveforms at applied voltage of 8 kV are shown in Figure 3. It can be seen from Figure 3 that each repetitive pulse appears in one division of oscilloscope screen, implying the duration of one cycle of waveform of 50 μS. Therefore, the pulse repetition frequency can be obtained 20000 Hz for the data shown in Figure 3. A good stability for discharge is also seen from the figure since the occurrence of voltage pulses and their corresponding current follows a logical and systematic process. The bipolar behavior of the the discharge current is also inferred from the data represented in Figure 3. The high voltage transformer was equipped with a diminishing system making it possible for applied current and voltage to be measured directly using an oscilloscope (HAMEG HM 203–7 Germany) without employing a high voltage probe. In order to evaluate the efficiency of the discharge reactor for decomposition of chloroform, we used specific input energy (SIE) as expressed by Eq. 1. The discharge power was calculated by integrating of the voltage and current products in a pulse time and then multiplying it by the pulse frequency as shown in Eq. 2.

1 $$ SIE\left(\raisebox{1ex}{$J$}\!\left/ \!\raisebox{-1ex}{$L$}\right.\right)=\frac{discharge\  power\left(\raisebox{1ex}{$J$}\!\left/ \!\raisebox{-1ex}{$S$}\right.\right)}{gas\  flow\  rate\left(\raisebox{1ex}{$L$}\!\left/ \!\raisebox{-1ex}{$S$}\right.\right)} $$

2 $$ Discharge\  power\left(\raisebox{1ex}{$J$}\!\left/ \!\raisebox{-1ex}{$S$}\right.\right)=\left[{\displaystyle \int \left|\;V(t)\times I(t)\right.}\left.dt\right|\right]\left(\raisebox{1ex}{$J$}\!\left/ \!\raisebox{-1ex}{$ Pulse$}\right.\right)\times Pulse\  frequency\left(\raisebox{1ex}{$ Pulse$}\!\left/ \!\raisebox{-1ex}{$S$}\right.\right) $$

### Gas analysis

The gas mixture of the outlet of the reactor was analyzed before and after the plasma catalytic process after having reached the steady-state conditions. Downstream products were collected into a gas-tight syringe through inline sampling ports and were injected into the gas chromatograph for analysis by using sample injection volume of 1 ml. The chloroform influent and effluent concentrations were averaged over three samples that were taken for each operating condition. When catalyst incorporated with the NTP, sampling was delayed until a stable breakthrough observed at the outlet. Gas analysis was conducted using a Varian 3800 GC equipped with a Saturn 2200 mass spectroscopy system (GC/MS). The GC was also equipped with a SGE capillary column with the inner diameter of 0.22 mm, film diameter of 0.25 μm, and length of 25 m. The supplying carrier gas was helium with a flow rate of 2 ml min^−1^. Temperature programming was as follows: 60°C for 1 min and then increased by 15°C min^−1^ up to 180°C and kept constant there for 1 min. NO and NO_2_ were measured using a NO_x_ analyzer (G750 polytector, Germany). Two single gas detectors were also employed directly in order to analyze CO (SGA91, U.K.) and CO_2_ (Testo535, Germany). Ozone was monitored applying both KI titration method and an ozone analyzer (EST-1510, USA) for high and low concentrations respectively. Furthermore, a thermohygrometer (Testoterm, GmbH & Co., Germany) was used to record the temperature and humidity of the system before and after the plasma process. The difference of chloroform concentration before and after the catalytic plasma process was our indicator to determine the decomposition rate, and chloroform removal efficiency (RE) was then calculated as the following (Eq. 3):

3 $$ RE\left(\%\right)=\left(1-\frac{{\left[ chloroform\right]}_{out}}{{\left[ chloroform\right]}_{in}}\right)\times 100 $$

Where [chloroform]_out_ and [chloroform]_in_ are the chloroform concentration in the effluent and influent gas respectively.

Also the selectivity towards CO_2_ (${S}_{C{O}_2}$) is given as (Eq. 4):

4 $$ {S}_{C{O}_2}\left(\%\right)=\frac{\left[C{O}_2\right]}{{\left[ chloroform\right]}_{in}\times RE}\times 100 $$

Where [CO_2_] is the CO_2_ concentration in the effluent gas after plasma and catalytic processes.

## Results and discussion

### Catalyst properties

**Figure 4:**
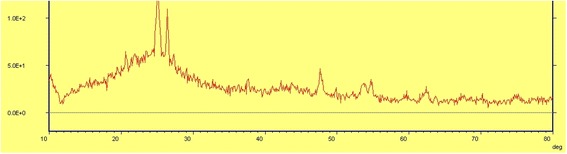
**XRD pattern of TiO**_**2**_ **nanoparticles coated on GAC.**

**Figure 5:**
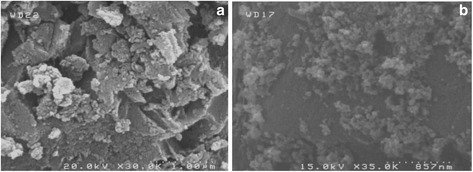
**SEM micrographs of (a) GAC, and (b) TiO**_**2**_ **/GAC.**

Both GAC and TiO_2_/GAC samples were subjected to XRD and SEM analysis. XRD and SEM profiles are shown in Figures 4 and 5, respectively. The only detected TiO_2_ phase by XRD analysis was anatase as can be seen in Figure 4, and no rutile and brookite phase were observed. Major XRD peaks for TiO_2_ particles were detected at 2θs of 25.1°, 47.6°, 53.7°, 54.7°, and 62.7°. A fairly high peak was also observed at 2θ = 26.4° for GAC itself. The average size of TiO_2_ particles was estimated to be in Nano scale, which was approved by SEM images, and Figure 5b shows that nanoparticles with a distribution diameter from ca. 50 nm up to several hundred nm were obtained. In our view, larger sizes of particles can be attributed to agglomeration, and despite the fact that the calcination temperature was relatively high around 300°C, agglomeration rate was also appeared to be significant. As can be seen in SEM images (Figure 5a and b), GAC had primarily a non-uniform, porous structure with small cavities and attached fine particles on its surface. It was then covered by TiO_2_ particles (Figure 5b), becoming smoother, more even, and uniform. The BET surface area was also determined with the N2 adsorption data in the relative pressure (P/P_0_) range of 0.05–0.577 K using a BELSORP-max nitrogen adsorption apparatus (Japan Inc.). The BET results are shown in Table 1. As can be seen in Table 1, the specific surface area was detected to be 969.1 and 935.2 m^2^ gr^−1^ for GAC and TiO_2_/GAC respectively. These findings revealed that the pore size of GAC was significantly affected by TiO_2_ coating and it decreased about 35 m^2^ gr^−1^ as TiO_2_ particles were covered on GAC surface. The total pore volume was also decreased from 0.5724 to 0.5453 cc gr^−1^ as the substrate surface covered by TiO_2_ nanoparticles. The reduction of surface area and total pore volume confirmed the SEM results. According to Figure 5, the deposition of TiO_2_ onto GAC pores led to disappearance of holes from its surface, leaving a lower level of porosity which implies a lower surface area.

**Table 1:** **The BET analysis data for GAC and TiO**_**2**_ **/GAC based on the nitrogen adsorption-desorption measurements**

**Sample name**	**GAC**	**TiO**_**2**_ **/GAC**
Specific surface area (m^2^g^−1^)	969.1	935.2
Total pore volume (cc g^−1^)	0.5724	0.5453

### The effect of pulse repetition frequency on removal efficiency

**Figure 6:**
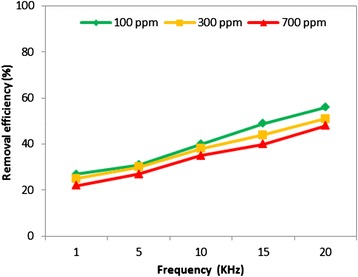
**The effect of frequency on chloroform removal efficiency with NTP-alone process (applied voltage of 18 kV).**

The design of power supply system enabled us to adjust the applied frequency up to 20 KHz during this study. In order to examine the effect of frequency on chloroform conversion, pulse repetition frequency was varied between 1 and 20 KHz while applied voltage fixed at 18 KV. Figure 6 shows this impact on the chloroform decomposition rate for different initial concentrations. As it is seen, RE was strongly influenced by increasing frequency for various concentrations, and the rate of conversion achieved 56% for 100 ppm concentration at a frequency of 20 KHz. The frequency increment gave same trend of decomposition with other concentrations (Figure 6). Our observations revealed that increasing frequency resulted in a change in the voltage and current waveforms by raising the number of pulses per one division on oscillogram, which makes the discharge power much stronger, and as a result, more energetic electrons are released, and the number of spontaneous collisions is increased. Consequently, the rate of reactions between chlorinated carbons and active species available in plasma region goes up which is expected. Due to the beneficial effect of pulsed repetition frequency, it was fixed at its highest value (20 KHz) in our study, and kept constant during all experiments as an optimal condition.

However, the effect of applied frequency on the conversion rate is rather controversial. It has been reported in some studies that a dramatic enhancement is achieved in removal efficiency with increasing frequency from 50 to 150 Hz and also over 1000 Hz [5,29,30]. Furthermore, Kogelschatz argued that the preferred working frequency for a DBD reactor is ca. 10 MHz [31]. By contrast, Oda et al. reported a reverse effect in the decomposition of CVOCs with increasing frequency and stated that the highest level of decomposition was achieved at a frequency of 100 Hz [32]. It should be noted here that the most suitable frequency depends largely on various plasma parameters, particularly the type of applied power supply system and reactor configuration.

### Plasma power effect on the decomposition of chloroform (SIE and catalyst introduction impact)

**Figure 7:**
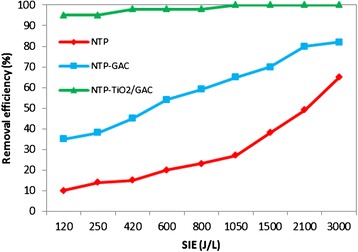
**Influence of SIE on chloroform removal efficiency (SIE 120–3000 J/L and 700 ppm of chloroform).**

SIE was varied between 120–3000 J L^−1^ during the present study by changing the applied voltage from 8 to 22 KV at a frequency of 20 kHz, and also gas flow rate from 0.2-1 L min^−1^. Figure 7 shows the conversion rate of 700 ppm of chloroform for SIE variation between 120 and 3000 J L^−1^. It is seen from Figure 7 that the conversion of chloroform was strongly influenced by the SIE. This dependency is observed in various conditions with and without catalyst, and is more noticeable for both NTP-alone and NTP-GAC systems. The trend is obviously upwards with NTP-alone process and the gradual rise in RE from 10 to approximately 30% is followed by a sharper increase at SIEs higher than 1000 J L^−1^ reaching 61% at highest SIE. It is also observed that the combination of NTP with TiO_2_/GAC is dependent on SIE to a lesser extent and even at SIE of 120 J L^−1^ the conversion rate rocketed to 95% with this coupling mode. This may be due to the fact that both TiO_2_ and GAC have strong catalytic activity and a hybrid of these seems to produce a dramatic synergistic effect. However, when TiO_2_/GAC was tested without NTP, no significant catalytic activity detected. Several studies have discussed the dependence of RE on energy density, supporting the observations made in the present study [33,34]. The main reason for this marked improvement may be the increased number of accelerated electrons due to higher input energy.

The incorporation of TiO_2_/GAC downstream of the NTP had a major effect on the chloroform conversion rate. 3 gram of TiO_2_/GAC was used each time until catalyst deactivation occurred. As can be seen in Figure 7 chloroform decomposition is strongly enhanced with NTP-catalyst hybrid system and RE of 100% achieved at SIEs higher than 1000 J L^−1^ as compared to NTP-alone for which RE was about 60% at highest possible SIE. At the same time, ozone was considerably decomposed over TiO_2_/GAC and reduced to less than 1 ppm in the effluent gas. These findings indicate that TiO_2_/GAC plays a prominent role in both chloroform and ozone destruction and that decomposition of ozone is highly beneficial for chloroform conversion. This is attributed not only to the GAC or TiO_2_ itself but also to the method of coating, because TiO_2_/GAC preparation with another impregnation technique, did not yield same results. In the presence of catalyst other harmful by-products along with ozone were also decreased significantly. For instance, NO_2_ that was 50 ppm for NTP-alone at SIE of 250 J L^−1^ was removed totally with NTP-TiO_2_/GAC system. The absence of NO was also observed with this coupling system while its amount was more than 80 ppm with the NTP-alone process. Because a catalytic activity was also expected for GAC, NTP was coupled with bare GAC in a series of our experiments. As it is seen from Figure 7, our expectation came true and chloroform conversion increased about 20% when GAC introduced downstream of the NTP. A marked decline was also observed in ozone concentration and it decreased to ca. 100 ppm at SIE of 3000 J L^−1^, whereas at the same SIE for NTP-alone it was as high as 700 ppm. NO, and NO_2_ levels, however, remained the same as for NTP-alone process.

Since the gas temperature ranged between 30–40°C downstream of the plasma and catalyst reactor, dissociation of ozone by heat is ruled out. If the difference in ozone level with NTP-GAC and NTP-TiO_2_/GAC is also considered, the role of TiO_2_/GAC catalyst is obviously determined in ozone decomposition. It is in good agreement in previous studies that TiO2 contributes to ozone decomposition significantly and the OH radicals are considerably improved when O_3_ passes over TiO_2_ in the presence of H_2_O [35-37]. OH radicals then can act as an oxidant agent in VOCs conversion as reported by Zhu et al. [37]. From these points we can conclude that TiO_2_/GAC catalyst leads chloroform and its intermediate products to further reactions which results in the greater production of water vapor and carbon oxides subsequently.

The abatement of other plasma generating by-products such as NO and NO_2_ over TiO_2_/GAC can also been considered as chloroform removal efficiency promoting according to reactions (1) and (2) [38,39]:

(1)

$$ N{O}_2+HC\to H{C}_{oxidized}+2 NO $$

(2)

$$ H{C}_{oxidized}+2 NO\to {N}_2+{H}_2O+C{O}_2 $$

Besides the removal of NOx, these redox reactions can be concluded as totally favorable reactions, since they contribute to the stable, safe, and desired end products. The lower level of CO which was obtained over TiO_2_/GAC and will be discussed further in the following sections may be explained by the reaction (3) that is another crucial role for the TiO_2_/GAC catalyst [40]. Simultaneous removal of unwanted by-products can be considered as one of the major advantageous of TiO_2_/GAC.

(3)

$$ 2 NO+2CO\to {N}_2+2C{O}_2 $$

### Effect of initial concentration

To investigate the influence of various concentrations on the performance of the reactor, initial concentration was varied between 100 and 700 ppm during this study. Table 2 shows RE for various concentrations with catalytic and non-catalytic plasma as a function of SIE. A higher amount of pollutant molecules causes a noticeable difference in gas composition in the plasma reactor, and the number of molecules available for decomposition grows per unit of discharge volume. This influences the energy density and also the quantities of active species produced in the plasma zone. Hence, the energy level that each individual molecule receives for the conversion decreases significantly which results in lower RE. This may be the reason why increasing initial concentration had a deleterious effect on chloroform conversion with NTP-alone system (Table 2). On the other hand, it seems that introducing more gaseous molecules into the plasma zone will generate a larger numbers of metastable and charged species especially when the feeding molecules are heavier and contain more atoms as in the case of toluene introduction. This effect reduced the voltage applied to the pin-to-plate reactor due to a corona-to-spark transition mode. With chloroform feeding, however, it did not affect the system even at higher concentrations (700 ppm). The effect of higher amounts of chloroform subjected to the reactor was not significant in the presence of TiO_2_/GAC. As seen from Table 2, total oxidation (RE of 100%) was achieved over TiO_2_/GAC at all SIEs when chloroform feeding was below 300 ppm, and RE dropped by only 5% with concentration of 700 ppm. A strong adsorption property of GAC is likely the reason of this better performance. For lower concentrations all of the species received from plasma medium can be adsorbed by GAC and hence higher residence time results in quite perfect catalytic reactions over TiO_2_ surface while with increasing the initial gas feeding the breakthrough rises gradually, causing some compounds to leave the catalyst bed unconverted. The effect of higher gas feeding was also obvious when toluene was introduced into the reactor. A competition between toluene and chloroform molecules was observed to be in favor of toluene and its total oxidation was occurred with SIEs higher than 1000 J L^−1^, while at the highest possible SIE (2100 J L^−1^) some amounts of chloroform still remained unchanged. This may also be due to the fast deactivation of catalyst resulting from numerous carbons released from toluene molecules. Our findings are in good agreement with the previous works [7,41,42]. However, Magureanu [12] reported a higher level of decomposition for increasing input concentration, believing that secondary reactions triggered by secondary radicals and ions resulting from the initial decomposition of CVOC molecules causes such effect.

**Table 2:** **Influence of initial concentration on chloroform removal efficiency as a function of SIE with NTP-alone and catalytic NTP**

**SIE**	**Removal efficiency (%)**							
	**NTP-alone**			**NTP-TiO**_**2**_ **/GAC**				
	**100 ppm**	**300 ppm**	**700 ppm**	**100 ppm**	**300 ppm**	**700 ppm**	**Cloroform + Toluene**	
							**Chloroform (300 ppm)**	**Toluene (100 ppm)**
120	15	12	10	97	97	95	92	94
250	20	18	14	100	100	95	92	94
420	22	20	15	100	100	96	93	95
600	30	24	20	100	100	96.5	93	95
800	36	29	23	100	100	97	94	98
1050	44	36	27	100	100	97	95	100
1500	58	44	38	100	100	98.5	95	100
2100	62	51	49	100	100	99	97	100
3000	69	63	61	100	100	99.5	-	-

### Selectivity towards CO_2_ and CO_x_

**Figure 8:**
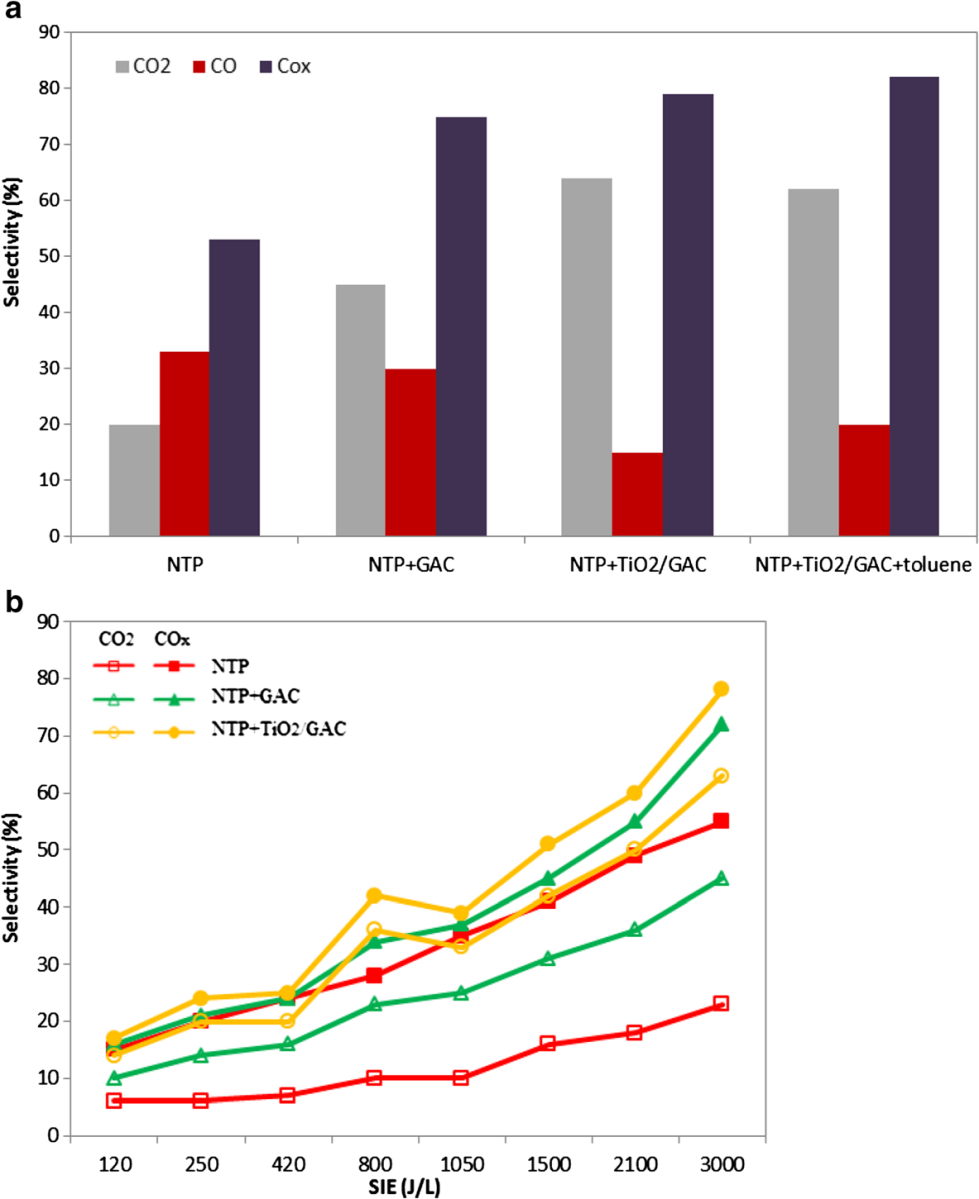
**CO, CO**_**2**_ **, and CO**_**x**_ **selectivity (a) at highest SIE (3000 J/L), and (b) as a function of SIE.**

While passing through the discharge zone and catalyst bed, the chloroform vapor decomposed into a mixture of CO_2_, CO, H_2_O, and some chlorinated products. Since CO_2_ is a desired final product, CO_2_ selectivity was calculated and plotted in Figure 8a and b along with CO_x_ selectivity which can represent the carbon balance if the amount of hydrocarbon by-products is negligible. Figure 8a presents the selectivity towards CO_2_, CO, and CO_x_ (CO_2_ + CO) for 700 ppm chloroform at SIE of 3000 J L^−1^. It is just evident from the figure that catalyst introduction influenced the products distribution effectively, and CO_2_ selectivity increased about 44% in the presence of TiO_2_/GAC, approaching almost 70%, whereas no result better than 20% was obtained with NTP-alone system. Although CO selectivity reduced substantially with TiO_2_/GAC, but selectivity towards CO_x_ still remained high due to the high level of CO_2_. Similarly, coupling NTP with bare GAC showed a much better activity with regard to CO_x_ selectivity, soaring up to 75%, while the amount of CO stayed approximately as high as that of the NTP-alone system. The most CO_x_ selectivity was observed for NTP-TiO_2_/GAC when toluene was added to the system, which was predictable due to the large quantities of carbon in toluene structure and also high quality of catalyst. Figure 8b shows CO_2_ and CO_x_ selectivity resulted from the decomposition of 100 ppm chloroform as a function of SIE. The trend is obviously upwards with increasing SIE under all experimental conditions as can be seen from Figure 8b. There was a marked improvement especially in CO_x_ selectivity over catalysts for SIEs above 1000 J L^−1^. From this result, it was recognized that ozone has a key role in improving the reactions towards both total oxidation and higher CO_x_ selectivity, because a dramatic increase was detected for ozone amount at these higher SIEs. Except for NTP-TiO_2_/GAC process, a large gap is evident between the CO_2_ and CO_x_ lines which is attributed to large quantity of CO produced under both NTP-alone and NTP-GAC conditions. No considerable influence of the chloroform initial concentration was observed on CO_2_ selectivity in the present study. Strong adsorption property of GAC may be the reason again. Nevertheless, Magureanu et al. [41] reported that the selectivity towards CO_2_ increases with lower gas flow rate and initial concentration, and argued that under these conditions higher residence time provides sufficient opportunity for the reactions to be carried out more efficiently. However, the dependence of CO_2_ selectivity on SIE as observed in the present study is in good agreement with the previous works [41,43].

### Mechanisms of chloroform oxidation and the formation of by-products

It was observed in several earlier studies that when the oxidation of chlorinated compounds occurs in a plasma reactor in an oxygen-rich environment such as air, a considerable amount of different chlorinated products can be produced, which could be more hazardous than the initial compound itself [44]. Among them, COCl_2_, Cl_2_, and CO seems to be the main harmful by-products [34]. The formation of products containing oxygen and chlorine is attributed to the reaction between chloride and oxygen atoms as described by Indarto et al., and Foglein et al. [23,24]. If the initial compound is chloroform, some amounts of HCl would also be produced along with COCl_2_ [23,44]:

(4)

$$ CHC{l}_3+O\to COC{l}_2+HCl $$

The reaction pathways of chloroform decomposition in a discharge medium near room temperature in the presence of oxygen have been studied in several earlier researches [11,24,45]. Based on these experimental works, one can conclude that many active radicals such as O^•^, OH^•^, Cl^•^, H^•^, CCl_3_^•^, CHCl_2_^•^ etc. are generated during the short initial step of decomposition resulting from the electron collisions with the existed molecules. The products of the fast reactions of this step are mainly dominated by both COCl_2_ and Cl_2_ through numerous reaction pathways [45]. These chlorinated intermediates participate in subsequent reactions with O_2_ to yield some peroxy radicals such as CHCl_2_O_2_, and CCl_3_O_2_, following by degradation into Oxy radicals, which in turn react with other radicals like Cl^•^ to form COCl_2_ according to Mok et al. [11]. A series of chain reactions with various paths occur at later stages leading to the formation of final products. There is no agreement among the various studies on final products. Foglein et al. found that the end products are stable and eco-friendly compounds such as H_2_O, CO_2_, and HCl [24]. However, considerable amounts of CO, and COCl_2_ were detected in the reactor outlet of other studies [11,23]. The production of Cl_2_ and CCl_4_ has also been noted in the literature [24,45].

**Figure 9:**
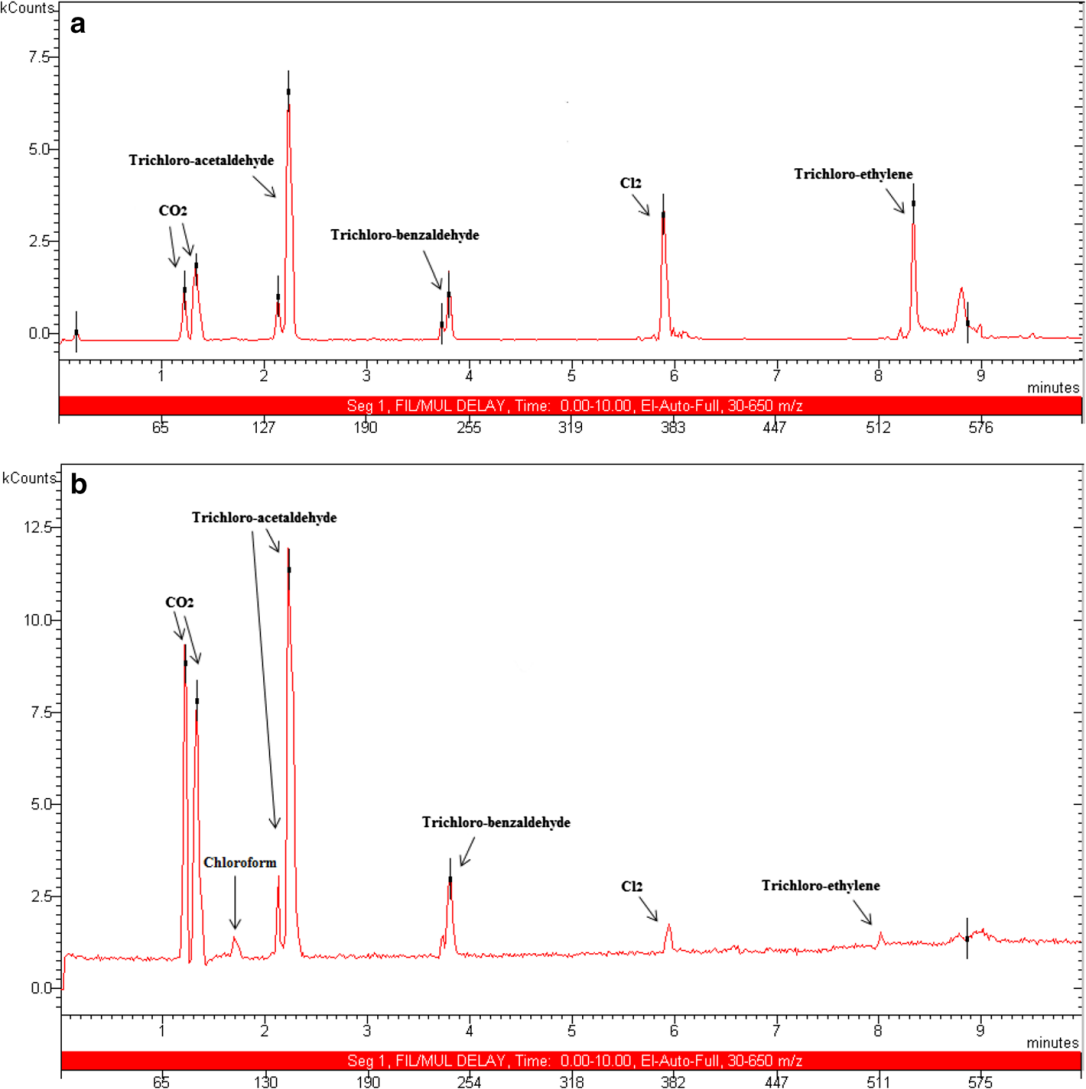
**GC/MS chromatogram of chloroform products (a) without toluene, (b) in the presence of toluene.**

Significant changes were detected for end products in the current study due to the catalyst and toluene incorporation. The final products detected during the present study under various conditions are listed in Table 3. In general, the decomposition products detected by GC/MS and other measuring apparatus during NTP-alone process included CO_2_, CO, H_2_O, Cl_2_, TCBA (trichloro-benzaldehyde), TCAA (trichloro-acetaldehyde), TCE (tri-chladf67oro-ethylene), NO, NO_2_, O_3_, and COCl_2_ as can be seen in Table 3. It is worth mentioning again that neither O_3_ nor NO_x_ was observed with NTP-TiO_2_/GAC while they were typical by-products in NTP-alone system. An interesting and favorable finding was also that no COCl_2_ was observed with NTP-TiO_2_/GAC with and without toluene addition. CO was detected with all conditions studied but its amount decreased over catalysts particularly with TiO_2_/GAC. During the oxidative decomposition of chloroform, the favorable products are CO_2_, H_2_O and desired chlorinated compounds such as HCl and chloroaldehides which are water-soluble and can be removed easily using a wet scrubber. Among the chlorinated products, Cl_2_ was the most abundant compound in the present study, followed by TCBA, TCAA, TCE, and COCl_2_ with plasma-alone process. In the presence of TiO_2_/GAC, however, COCl_2_ was totally removed and the values of others changed dramatically. Figure 9a represents a GC/MS chromatogram for 300 ppm of chloroform at SIE of 2100 J L^−1^ with NTP-TiO_2_/GAC. It is clearly evident from Figure 9a that the main chlorinated by-products include TCAA, Cl_2_, as well as TCE, which was followed by TCBA.

**Table 3:** **Main components of chloroform decomposition detected by analyzing apparatus**

**Compounds**	**m/z**	**NTP-alone**	**NTP-TiO**_**2**_ **/GAC**	**NTP-TiO**_**2**_ **/GAC + toluene**
CO_2_	40	*√*	*√*	*√*
CO	-	*√*	*√*	*√*
H_2_O	-	*√*	*√*	*√*
Cl_2_	74	*√*	*√*	*√*
TCBA	77-209	*√*	*√*	*√*
TCAA	148	*√*	*√*	*√*
TCE	97	*√*	*√*	*√*
NO	-	*√*	-	-
NO_2_	-	*√*	-	-
O_3_	-	*√*	-	-
COCl_2_	98	*√*	-	-
HCl	-	-	-	-

As mentioned earlier and represented in reaction (9), due to the great ability of TiO_2_/GAC for decomposition of ozone, the production of O radicals is highly appreciated. Therefore, the COCl_2_ removal over catalyst surface may be attributed to the reaction between COCl_2_ and provided O radicals [46]:

(5)

$$ COC{l}_2+{O}^{\bullet}\to C{O}_2+C{l}_2 $$

As a result of this reaction, the selectivity towards CO_2_ and Cl_2_ can be considerably improved as observed with the NTP-TiO_2_/GAC system.

We introduced a hydrogen-rich compound i.e. toluene into the gas stream to shift the reactions to HCl production in the present study. Two main reasons supported this Idea. First, when toluene introduces into the plasma medium, numerous H^•^ radicals are predicted to generate along with O^•^ and Cl^•^, and the number of spontaneous collisions between these radicals is therefore enhanced significantly, resulting in more HCl and H_2_O production. Second, there is a strong electronegativity between H^•^, locating in the extreme left of the periodic table, and Cl^•^, in the extreme right, offering more HCl production. Foglein et al. [24] also noticed that the presence of hydrogen results in a large number of active radicals such as OH^•^, and HO_2_^•^ that lead to the subsequent decomposition of COCl_2_ so fast that leaves it as an intermediate product. As mentioned earlier and shown in Table 3, toluene was converted totally itself but its presence decreased the decomposition of chloroform for the same SIE and initial concentration levels that were tested previously. It is likely related to consumption or deactivation of some radical intermediates present in the NTP reactor by toluene molecules. Table 3 represents the behavior of NTP-TiO_2_/GAC system in the presence of toluene in relation to final products. The amount of H_2_O was enhanced significantly after toluene introduction, and relative humidity reached up to 65% in the effluent gas at SIE of 2100 J L^−1^. From these results, OH and HO_2_ radicals may be appeared as two major active species generated under toluene feeding conditions but their amount seems to not exceeding that of ozone for which the concentration was still below 1 ppm downstream of the catalyst. Here, a synergy can be recognized between H radicals provided from toluene molecules and ozone that can act as an electron acceptor and donor over catalyst surface to generate OH^•^ and HO_2_^•^ according to Einaga and Ogata [47]:

(6)

$$ {O}_3+{e}^{-}\to {O_3}^{\bullet -} $$

(7)

$$ {H}^{+}+{O_3}^{\bullet -}\to H{O_3}^{\bullet } $$

(8)

$$ H{O_3}^{\bullet}\to {O}_2+O{H}^{\bullet } $$

(9)

$$ {O}_3+{e}^{-}\to {O}_2+{O}^{\bullet } $$

(10)

$$ {O}_3+O{H}^{\bullet}\to H{O_2}^{\bullet }+{O}_2 $$

(11)

$$ {O}^{\bullet }+H{O}_2\to 2O{H}^{\bullet } $$

Although no peak was again observed for HCl on mass spectrograms, but a high HCl concentrations were detected using gas detector tubes. HCl was not possible to determine using GC/MS resulting from the interferences between hydrogen chloride and other chlorinated products [48]. However, using hydrogen chloride detector tubes, a favorable selectivity towards HCl was noticed. It seems that addition of hydrogen-rich molecules such as toluene shifts the reaction in both plasma zone and catalyst reactor and causes some reaction pathways to be dominant and contributes to the formation of more H_2_O, and HCl [47,48].

(12)

$$ O{H}^{\bullet }+CHC{l}_3\to {H}_2O+CC{l}_3 $$

(13)

$$ COC{l}_2+{H}^{\bullet}\to COC{l}^{\bullet }+HCl $$

(14)

$$ HCOCl+C{l}^{\bullet}\to ClC{O}^{\bullet }+HCl $$

During the toluene addition, a significant decline was also detected for Cl_2_ and TCE, for which the total removal was not achieved but their peak area became negligible. The disappeared Cl_2_ may be responsible for the further HCl production in the reaction with water vapor over TiO_2_/GAC [49]:

(15)

$$ {H}_2O+C{l}_2\iff 2HCl+\frac{1}{2}{O}_2 $$

Two chlorinated by-products that were observed to be increased in the presence of toluene were TCAA and TCBA. These results can be clearly seen in Figure 9b. It is evident from Figure 9b that with toluene feeding, the level of CO_2_, TCAA, as well as TCBA increased obviously as compared for chloroform introduction alone (Figure 9a). These findings reveal that toluene addition is highly beneficial in relation to products distribution towards the less harmful by-products. Although these products are heavy aldehyde compounds but they are highly water-soluble and can be totally removed utilizing a wet scrubber downstream of the catalyst reactor. With toluene addition, these desirable results were obtained along with some limitations. First, the extent of other undesirable by-product i.e. CO also went up from less than 15 ppm to ca. 160 ppm for the highest SIE and Figure 8 shows that toluene introduction increased the selectivity towards CO about 5% which was expected due to higher quantities of C- atoms in the toluene structure. Adding hydrogen gas or H_2_O may be a good alternative option in this regard. Second, because of higher concentrations and a major difference in the gas composition, the voltage at which corona-to-spark transition occurred, decreased substantially from 22 to 18 KV as toluene fed to the reactor and therefore supplying less energy was achievable. Another reactor configuration combined with other catalysts in the presence of toluene and H_2_O is under investigation in order to overcome some of these limitations.

## Conclusions

Influence of SIE, initial concentration, and toluene addition on chloroform removal efficiency was investigated in a catalytic plasma pin-to-plate corona discharge reactor. A much better performance was observed for hybrid process in both removal efficiency and COx selectivity compared to NTP-alone system. Ozone was noticed to have a powerful effect on chloroform destruction on TiO_2_/GAC surface, and realized to play a key role in producing some active radicals which are helpful to remove hazardous by-products. Strong adsorption property of GAC affected the catalytic activity dramatically. Even though the toluene introduction was highly beneficial for shifting the reactions towards less harmful products, it slightly reduced the chloroform removal efficiency.
